# Prevalence and patterns of multi-morbidity in the productive age group of 30-69 years: A cross-sectional study in Pathanamthitta District, Kerala.

**DOI:** 10.12688/wellcomeopenres.16326.2

**Published:** 2020-12-15

**Authors:** Rohini C, Panniyammakal Jeemon

**Affiliations:** 1 Achutha Menon Centre for Health Science Studies, Sree Chitra Tirunal Institute of Medical Sciences and Technology, Trivandrum, Kerala, 695011, India

**Keywords:** Multi-morbidity, cross-sectional, household, rural, India, pattern

## Abstract

**Background**: Multi-morbidity is the coexistence of multiple chronic conditions in individuals. With advancing epidemiological and demographic transitions, the burden of multi-morbidity is expected to increase India.

**Methods**: A cross-sectional representative survey was conducted among 410 participants aged 30-69 years in Pathanamthitta District, Kerala to assess the prevalence of multi-morbidity. A multi-stage cluster sampling method was employed to identify households for the survey. We interviewed all eligible participants in the selected households. A structured interview schedule was used to assess socio-demographic variables, behavioral risk factors and prevailing clinical conditions. We used the PHQ-9 questionnaire for depression screening. Further, we conducted active measurements of both blood sugar and blood pressure. Multiple logistic regression was used to identify variables associated with multi-morbidity.

**Results**: Overall, the prevalence of multi-morbidity was 45.4% (95% CI: 40.5-50.3%). Nearly a quarter of the study participants (25.4%) reported only one chronic condition (21.3-29.9%). Further, 30.7% (26.3-35.5), 10.7% (7.9-14.2), 3.7% (2.1-6.0) and 0.2% reported two, three, four and five chronic conditions, respectively. At least one person with multi-morbidity was present in around seven out of ten households (72%, 95% CI: 65-78%). Further, one in five households (22%, 95% CI: 16.7-28.9%) reported more than one person with multi-morbidity. Diabetes and hypertension was the most frequent dyad (30.9%, 95% CI: 26.5-35.7%), followed by hypertension and depression (7.8%, 95% CI: 5.5-10.9%). Diabetes, hypertension and ischemic heart disease was the common triad in males (8.5%, 95% CI: 4.8-14.1%), while it was diabetes, hypertension and depression (6.9%, 95% CI: 4.2-11.1%) in females. Age, sex, and employment status were associated with multi-morbidity.

**Conclusion**: Multi-morbidity is prevalent in one of two participants in the productive age group of 30-69 years. Further, seven of ten households have at least one person with multi-morbidity. The high burden of multi-morbidity calls for integrated management strategies for multiple chronic conditions.

## Introduction

Globally, several countries are going through an epidemiological transition in which degenerative diseases are displacing infections and nutritional disorders as the major cause of mortality and morbidity ^1^. An increasing trend of life expectancy due to good living conditions, increased income per capita, advances in health care and education are reported globally ^2, 3^. However, the added years of life due to increase in life expectancy are often complicated by poor health and disability, resulting from the high burden of chronic diseases, especially non-communicable diseases (NCDs) ^4^. Available evidence suggests that the majority of patients diagnosed with chronic diseases have more than one chronic co-existing condition ^5^. Co-existence of more than one chronic condition in the same individual is known as multi-morbidity ^6^.

Prevalence estimates of multi-morbidity vary widely depending on method of data collection ^4^, age of the population studied, definition of chronic condition, definition of multi-morbidity, and number and types of chronic conditions included in the analysis. In addition to the complexities related to the definition, there is a lack of an explicit validated tool to measure the components of multi-morbidity uniformly for reporting the magnitude of diseases ^7^. However, available evidence indicates that prevalence, pattern and complexity associated with multiple chronic conditions have been increasing over the years ^8^.

The increasing burden of multi-morbidity may cause considerable financial burden, especially in low- and middle-income countries (LMICs). If preventive and management strategies are not integrated and coordinated across multiple chronic conditions, it can lead to duplication and health system inefficiency. Data from LMICs on multi-morbidity are very limited. Further, India does not have population level estimates on multi-morbidity. Given the rising burden of NCDs in India, with the onset of disease at least a decade earlier than their Western counterparts ^9^, it is important to characterize the multi-morbidity pattern in this population.

The state of Kerala in India is in an advanced stage of epidemiological transition as compared to other states in India ^10, 11^. Therefore, it is highly likely that the prevalence of multi-morbidity will be higher in Kerala. However, very limited data on prevalence of multi-morbidity are available from Kerala. A better understanding of the epidemiology of multi-morbidity is crucial for re-organization of health care services to provide integrated care for multiple chronic conditions. The objective of our study was to assess the community level prevalence and pattern of multi-morbidity in Kerala.

## Methods

### Ethical statement

The Institutional Ethics Committee (IEC) of Sree Chitra Tirunal Institute for Medical Sciences and Technology, Trivandrum approved the study (SCT/IEC/1455/NOV-2019). The questionnaire interview and all measurements were conducted after obtaining a written informed consent from each study participant. Privacy was ensured during the time of interview and confidentiality of all the information collected was maintained. The participants agreed to report or publish the data collected during the study except any information that could lead to the identification of any individual, by signing a consent form prepared in local language. The participants had the freedom to refuse participation at the beginning or during any stage of data collection.

### Study design

A cross-sectional survey was conducted in Pathanamthitta district of Kerala, India using a structured interview schedule.

### Study setting

Pathanamthitta is a southern district of Kerala with the highest proportion of elderly population (18%), lowest total fertility rate (1.3) ^12^ and highest literacy rate (96.9%) ^13^. The population size of Pathanamthitta district is close to 1.2 million as per the 2011 census ^14^. A high prevalence of diabetes is also noted in Pathanamthitta district ^15^.

### Study population

We included eligible participants aged 30 to 69 years. The participants were residents of rural areas of Pathanamthitta district for a minimum period of one year. Those who did not give informed consent, were physically or mentally not in a condition to answer the questions and/or undergo clinical measurements as part of the study, or were pregnant or lactating within six weeks post-partum were excluded. All eligible participants in a selected household who satisfied the inclusion criteria were included in the study.

There are eight community development blocks (CDB) in Pathanamthitta district. The CDBs were the primary sampling units. Of these eight CDBs, three were randomly selected by simple random sampling. We used computer generated random numbers for the random selection of CDBs. In the second stage, four panchayats were selected from each CDB using computer generated random numbers (a total 12 of Panchayats were selected from three blocks). From these 12 panchayats, one ward each was randomly selected using computer generated random numbers. Further, 16 to 18 alternate houses were visited in each ward. One of the authors (RC) was responsible for the selection of households and participants for the study. After locating the center and the main junction of the ward, by pen rotation, the first household was identified. Subsequently, every alternate household was visited. In case the selected house was empty or locked, the next nearest one was visited. All the houses visited were on the right hand side of the road. All the eligible participants in the selected households were interviewed. In case any of the potential participants were not present in the house during the time of interview, one more attempt was made at a later time point.

### Data collection

A medically qualified primary care doctor (RC) conducted the data collection from all participants. A community health worker (ASHA) assisted RC in the survey. A structured interview schedule ^16^ was used to assess variables such as socio-demographic factors, behavioral risk factors and prevailing clinical conditions. We used the translated patient health questionnaire (PHQ-9) in *Malayalam* (local language in the state of Kerala) for screening of depression. Random capillary blood sugar (Onetouch Verio Flex Meter) and blood pressure (Omron Blood pressure monitor-upper arm BP7100) measurements were also undertaken. Three readings were recorded at five minutes intervals in the non-dominant arm by getting the participant to sit comfortably for five minutes and keeping the machine at the same level of his/her heart. The average of the three values was calculated and considered as the blood pressure of the participant. Elderly (above 60 years) participants were asked about their living arrangements. Females were asked about their history of menopause. The period of data collection was from 01.01.2020 to 28.02.2020.

### Definitions

The operational definition of ‘multi-morbidity’ was ‘co-existence of more than one of the 11 listed chronic conditions in the same individual’. Hypertension was defined as per Joint National Committee-7 guidelines ^17^. Depression was defined as a PHQ-9 score of 10 or above. Diabetes mellitus was defined as a random capillary blood glucose value above 140mg/dl. The remaining conditions viz ischemic heart disease, heart failure, stroke, chronic kidney disease, chronic obstructive airway disease, arthritis, thyroid hormone disorders and cancer were self-reported by the participants and verified with corresponding patient-held medical records, which were present with them. The patient-held medical records included consultation details, diagnostic details and treatment details. Those who ever used any form of tobacco were defined as tobacco users and those who ever used alcohol were defined as alcohol users. Recommended level of physical activity was defined as walking/jogging/engaging in sport for at least 30 minutes a day on five days in a week. A household was defined as a group of persons who live together and share a common kitchen. The age of starting formal education in the state of Kerala is six years. Low education group were those who studied up to 7 ^th^ standard. Those who studied above 7 ^th^ standard were defined as high education group.

### Sample size

A prevalence of 30% was anticipated ^18^ and the precision assumed was 6%. A design effect of 1.5 was assumed, as we employed a multi-stage cluster sampling to identify the participants. We accounted for 10% attrition. The sample size required was estimated as 403 and it was rounded off to 410.

### Data management and data analysis

Data were collected using paper forms and later entered into Microsoft Excel 2013 (v 15.0) sheets ^16^. Data cleaning and data analysis were done using R software version 3.6.3 ^19^. We used the R packages “summarytools’ and ‘ggplot2’. Continuous variables were presented as mean and standard deviation and categorical variables as proportions. The overall prevalence and prevalence across socio-demographic variables were calculated with 95% confidence intervals (CIs). Logistic regression analyses were performed to estimate odds ratios (ORs) of various socio-demographic factors associated with multi-morbidity. We present both un-adjusted and adjusted multi-variate model (with sandwich variance estimator) results.

## Results

### General characteristics

Out of the 430 participants approached, we collected data from 410 participants (95% response rate) ^16^. The mean age of the study population was 53 (SD=11.7) years ( Table 1). Three of five participants in the study population were females (59.8%). Females were younger (mean=52, SD=12.2 years) compared to males (mean=55, SD=10.7 years). Nearly one-third of the participants (32%) reported education up to seventh standard. Nearly half (49%) of the study population were homemakers (49%). One of five participants (20%) were ever users of tobacco. Alcohol use (ever use) was prevalent in 14.6% of the study population. Recommended level of physical activity was reported in one of five participants (20%).

**Table 1.** General characteristics of the study population.

Characteristics	Females N=245	Males N=165	Total N=410
**Age (mean, SD)**	51.9 (12.2)	55.0 (10.7)	53.2 (11.7)
**Age group, n (%)**			
30-39	48 (19.6)	15 (9.1)	63 (15.4)
40-49	59 (24.1)	37 (22.4)	96 (23.4)
50-59	53 (21.6)	43 (26.1)	96 (23.4)
60-69	85 (34.7)	70 (42.4)	155 (37.8)
**Religion, n (%)**			
Hindu	131(53.5)	92 (55.8)	223 (54.4)
Christian	110 (44.9)	72 (43.6)	182 (44.4)
Muslim	4 (1.6)	1 (0.6)	5 (1.2)
**Marital status, n (%)**			
Married	192 (78.4)	152 (92.1)	344 (83.9)
Divorced/separated	5 (2.0)	2 (1.2)	7 (1.7)
Widowed	43 (17.6)	4 (2.4)	47 (11.5)
Unmarried	5 (2.0)	7 (4.2)	12 (2.9)
**Education, n (%)**			
No schooling	1 (0.4)	1 (0.6)	2 (0.5)
1st-7th standard	85 (34.7)	45 (27.3)	130 (31.7)
8th -10th standard	109 (44.5)	75 (45.5)	184 (44.9)
Higher secondary	17 (6.9)	29 (17.6)	46 (11.2)
Graduation and above	33 (13.5)	15 (9.1)	48 (11.7)
**Employment, n (%)**			
Daily wager/self-employed	24(9.8)	89 (53.9)	113(27.6)
salaried	12 (4.9)	10 (6.1)	22 (5.4)
Unemployed	209(85.3)	66 (40)	275 (67.1)
**Monthly income, n (%)**			
>=25000 INR	51 (20.8)	33 (20.0)	84 (20.5)
<25000 INR	194 (79.2)	132 (80.0)	326 (79.5)
**Ever used tobacco, n (%)**	1 (0.4)	81 (49.1)	82 (20.0)
**Ever used alcohol, n (%)**	0 (0.0)	60 (36.4)	60 (14.6)
**Recommended physical** **activity, n (%)**	28 (11.4)	54 (32.7)	82 (20.0)

INR, Indian Rupee; SD, standard deviation.

### Prevalence of multi-morbidity

**Figure 1.**
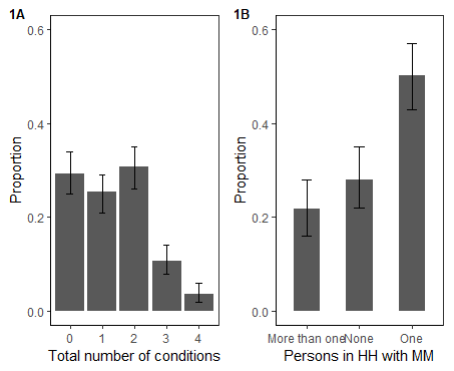
**A**) Distribution of morbidities among the participants **B**) Proportion of households (HH) with and without multi-morbidity (MM).

More than a quarter (29.3%) of the study participants did not report any one of the 11 included chronic conditions ( Figure 1A). Nearly a quarter of the study participants (25.4%) reported only one condition (95% CI: 21.3-29.9). Further, 30.7% (95% CI: 26.3-35.5), 10.7% (95% CI: 7.9-14.2), 3.7% (95% CI: 2.1-6.0) and 0.2% reported two, three, four and five co-existing conditions, respectively ( Figure 1A). Overall, 45.4% (95% CI: 40.5-50.3) of the study population reported two or more conditions or multi-morbidity.

Around seven in ten households (72%) reported at least one person in the household with multi-morbidity. Additionally, one in five households (22%) reported more than one person with multi-morbidity ( Figure 1B). Overall, 28% of the households did not report any one with multi-morbidity.

### Pattern of multi-morbidity

**Figure 2.**
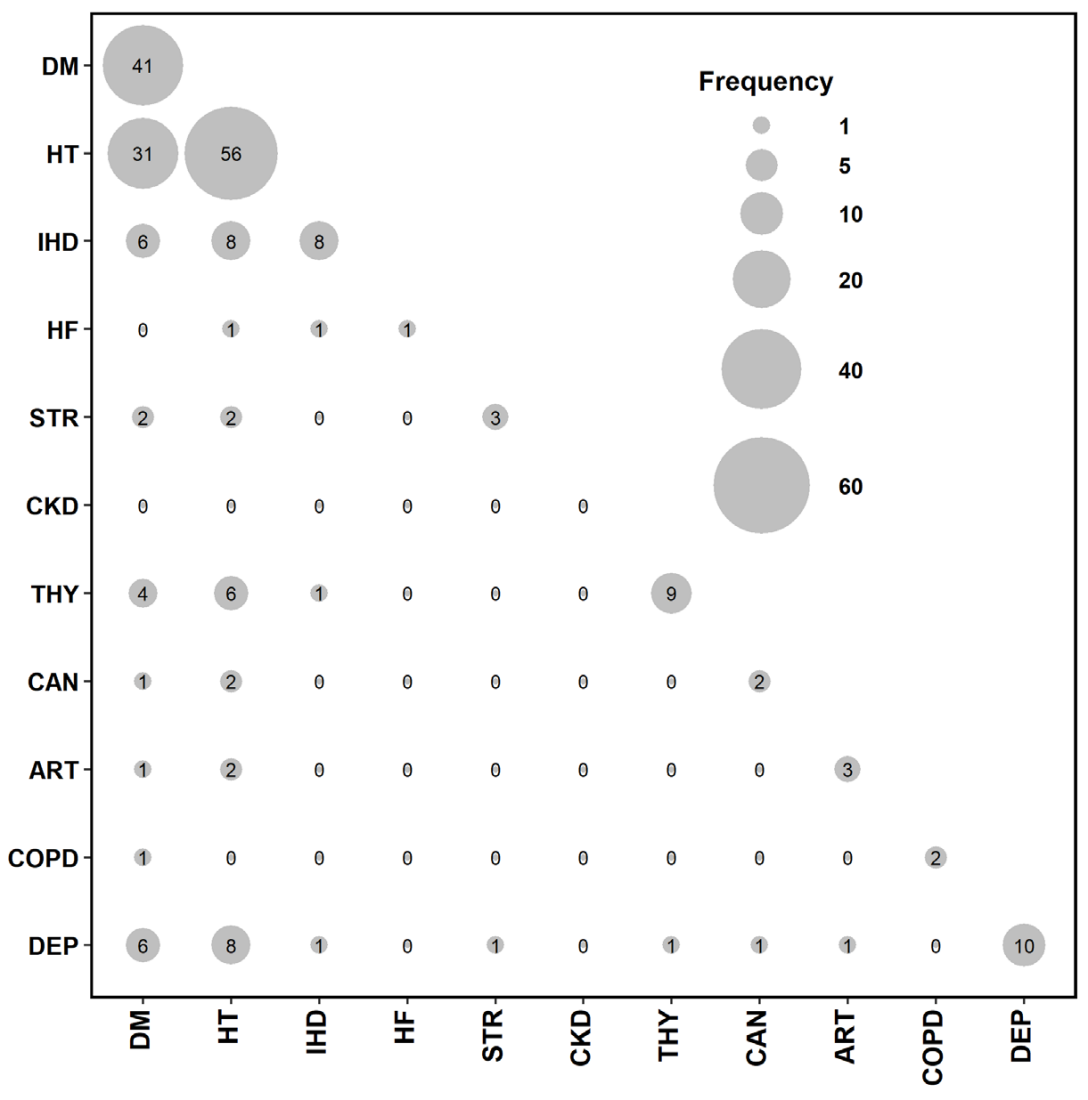
Pattern of co-existence of conditions. DM, diabetes mellitus; HT, hypertension; IHD, ischemic heart disease; HF, heart failure; STR, stroke; CKD, chronic kidney diseases; THY, thyroid hormone disorders; CAN, cancer; ART, arthritis; COPD, chronic obstructive airway disease; DEP, depression.

We present common patterns of multi-morbidity in Table 2. Diabetes and hypertension were the most frequently co-existing conditions (31%, 95% CI: 26.5-35.7%) ( Figure 2). The second most common pair in females was hypertension-depression (11%, 95% CI: 7.8-16.2%), followed by diabetes-depression (10%, 95% CI: 6.5-14.4%). However, in males it was hypertension-ischemic heart disease (11.5%, 95% CI: 7.2-17.6%) followed by diabetes-ischemic heart disease (8%, 95% CI: 4.9-14.1%).

**Table 2.** Common pairs of chronic conditions (pattern of multi-morbidity) among the study population.

Common dyads, n (%)	Total (N=410)	Males (N=165)	Females (N=245)
Diabetes – Hypertension	127 (30.9)	51 (30.9)	76 (31.0)
Hypertension – Depression	32 (7.8)	4 (2.4)	28 (11.4)
Hypertension – Ischemic heart disease	31 (7.5)	19 (11.5)	12 (4.9)
Diabetes – Depression	26 (6.3)	2 (1.2)	24 (9.8)
Diabetes – Ischemic heart disease	23 (5.6)	14 (8.4)	9 (3.7)
Hypertension – Thyroid hormone disorders	23 (5.6)	2 (1.2)	21 (8.6)
Diabetes – Thyroid hormone disorders	16 (3.9)	2 (1.2)	14 (5.7)
Hypertension – Stroke	10 (2.4)	7 (4.2)	3 (1.2)
**Common triads, n (%)**			
Diabetes – Hypertension – Ischemic heart disease	23 (5.6)	14 (8.5)	9 (3.6)
Diabetes – Hypertension – Depression	19 (4.6)	2 (1.2)	17 (6.9)
Diabetes – Hypertension – Thyroid hormone disorders	11 (2.6)	1 (0.6)	10 (4.1)
Diabetes – Hypertension – Stroke	6 (1.4)	3 (1.8)	3 (1.2)
Diabetes – Thyroid hormone disorders – Depression	4 (0.9)	0 (0.0)	4 (1.6)

### Multi-morbidity in sub-groups of population

The prevalence of multi-morbidity increased from 3.2% in the 30–39 age group to 69.7% in the 60–69 age group (p<0.001). More than half of the study participants (53.1%) in the 50–59 age group reported multi-morbidity ( Table 3). In the multiple logistic regression analysis, a 10% increase in propensity for multi-morbidity with every one year increase in age was observed (OR=1.1; 95% CI: 1.1-1.2, p<0.001). The prevalence of multi-morbidity was relatively higher in females (48.2%) as compared to males (41.2%). Similarly, the odds for multi-morbidity were lower in males as compared to females (OR=0.4, 95% CI: 0.2-0.8, p=.015). The low education group had a higher prevalence (53%) of multi-morbidity than the high education group (41.7%) (p=0.04). However, in the multi-variate model, after accounting for other socio-demographic variables, participants in the low education category had lower odds of multi-morbidity as compared to individuals in the high education category (OR=0.40; 95% CI: 0.2-0.7, p<.001). The unemployed group had a higher prevalence of multi-morbidity as compared to others (p<0.001). Marital status, income, alcohol and tobacco intake in males and living arrangement of elderly did not influence the propensity for multi-morbidity.

**Table 3.** Prevalence of multi-morbidity (MM) according to socio-demographic characteristics.

	No MM N=224	MM N=186	Crude OR	Adjusted OR *	*P* value
**Age per one year increase**			1.1 (1.08-1.13)	1.1 (1.1-1.2)	<0.001
**Age groups, n (%)**				Not included	
30-39	61 (96.8)	2 (3.2)	Reference		
40-49	71 (74)	25 (26)	10.7 (2.4-47.2)		
50-59	45 (46.9)	51 (53.1)	34.6 (7.9-149.4)		
60-69	47 (30.3)	108 (69.7)	70.1 (16.4-298.6)		
**Gender, n (%)**					
Female	127 (51.8)	118 (48.2)	Reference		
Male	97 (58.8)	68 (41.2)	0.7 (0.5-1.1)	0.4 (0.2-0.8)	0.015
**Marital status, n (%)**					
Currently not married ***	25 (37.9)	41 (62.1)	Reference		
Married	199 (57.8)	145 (42.2)	0.4 (0.2-0.7)	0.7 (0.3-1.5)	0.324
**Education, n (%)**					
Above primary	162 (58.3)	116 (41.7)	Reference		
Primary	62 (47.0)	70 (53.0)	1.6 (1.03-2.4)	0.4 (0.2-0.7)	<0.001
**Employment, n (%)**					
Daily wage/self-employed	81 (71.7)	32 (28.3)	Reference		
Unemployed	126 (45.8)	149 (54.2)	2.9 (1.8-4.8)	1.9 (1.03-3.6)	0.041
Salaried	17 (77.3)	5 (22.7)	0.7 (0.2-2.2)	0.9 (0.3-3.1)	0.529
**Income, n (%)**				*	
High income	38 (45.2)	46 (54.8)	Reference		
Low income	186 (57.1)	140 (42.9)	0.6 (0.4-1.0)		
Ever had tobacco **, n (%)				*	
Yes	49 (60.5)	32 (39.5)	0.7 (0.4-1.2)		
No	48 (57.1)	36 (42.9)	Reference		
Ever had alcohol **, n (%)				*	
Yes	36 (60)	24 (40)	0.7 (0.4-1.3)		
No	61 (58.1)	44 (41.9)	Reference		
**Living arrangement-elderly, n (%)**				*	
With spouse (n=116)	42 (36.2)	74 (63.8)	0.5 (0.2-1.1)		
Without spouse(n=39)	8 (20.5)	31 (79.5)	Reference		

*Only variables that showed statistical significance (p<0.05) in unadjusted analyses were entered into adjusted multi-variate analysis. **Analysis was done only for male participants as females did not report alcohol or tobacco use. MM, multi-morbidity; OR, odds ratio. ***Currently not married means either unmarried or divorced/separated or widowed

Among the 245 female participants, 63.3% had reached menopause. Among those who had reached menopause, the prevalence of multi-morbidity was 66.5%. However, the prevalence of multi-morbidity was 16.7% among females who did had not reached menopause. 

## Discussion

### Highlights

Multi-morbidity, the co-existence of multiple chronic conditions in the same individual, is prevalent in one of two adult individuals in the age group of 30–69 years in Pathanamthitta district of Kerala. The prevalence of multi-morbidity increases with advancing age. We also document that seven in ten households have at least one individual with multi-morbidity. Further, one in five households report more than one person with multi-morbidity. Diabetes and hypertension are the most common co-existing conditions. However, we find differences in the second and third most common dyads and triads of co-existing chronic conditions between males and females. Multi-morbidity assessment in management of chronic conditions is essential given its high prevalence and the differences in health care utilization and quality of life in individuals with different combinations of co-existing chronic conditions ^20^.

### Comparison of multi-morbidity prevalence with other studies

Direct comparison of prevalence of multi-morbidity across studies is almost impossible due to varying definitions of multi-morbidity, methods of data collection and the number of conditions included. In a systematic review, the prevalence of multi-morbidity ranged from 4.5% to 83% in the South Asian population ^21^. Additionally, in a previous study conducted in Kerala among the age group of 45 years and above, the prevalence of multi-morbidity was substantially lower than our study estimates ^22^. Most of the studies on multi-morbidity in India and other LMICs rely on self-reported data on chronic conditions from the participants and are often subjected to several biases. In our study, we incorporated active measurements for diabetes, hypertension and depression assessment. Further, the medical records of the participants were verified by a medical doctor to confirm the self-reported data on other chronic conditions. The number of chronic conditions included in our assessment for multi-morbidity was also higher than other studies conducted in LMICs.

### Multi-morbidity assessment at the household level

The household is an important unit of intervention for chronic disease prevention and control. Although the data on the prevalence of multi-morbidity at the household level are limited, available data suggest strong concordance of chronic conditions within the co-residing adults ^23^. Existence of at least one person with a chronic disease among seven out of ten households in Kerala is alarming. Further, multi-morbidity was prevalent in more than one individual in one of five households. The alarming prevalence at the household level calls for innovative models for prevention and control of multi-morbidity. Family-based models such as the Programme of Lifestyle Intervention in Families (PROLIFIC study) ^24^ may be more appropriate to reduce the burden and progression of multi-morbidity in LMICs. A family centered approach for lifestyle changes and self-care for cardiovascular risk reduction, and involvement of non-physician health workers for care-coordination were the key strategies in the PROLIFIC study. It is envisaged that in a family focused approach, the proposed lifestyle changes and self-care strategies are more achievable and sustainable for both the individuals and their family members ^25^.

### Multi-morbidity prevalence in sub-groups and their implications

The prevalence of multi-morbidity increases with age. In our study, the highest prevalence of multi-morbidity was among the age group of 60–69 years. Similar findings were reported in studies from other countries ^21, 26– 28^. Although seven out of ten participants in the 60–69 age group reported multiple chronic conditions in our study, it was evident that multi-morbidity is a substantial problem even in the younger population. The relatively high burden of multi-morbidity in the most productive age group is a cause for concern as it has serious implications in terms of productivity loss, higher health care utilization ^20^ and health expenditure ^29^.

In our study the prevalence of multi-morbidity among females was higher in comparison to males. Several other studies from India and other Asian countries reported a higher prevalence of multi-morbidity in females ^22, 29, 30^. Further, high prevalence of multi-morbidity among unmarried, divorced/separated/widowed individuals are also reported in other studies ^31, 32^. Gender differences in multi-morbidity pattern and care utilization should be explored in detail in future studies.

There is contrasting evidence regarding the association of education or socio-economic status with multi-morbidity. Data from high-income countries suggest an inverse relationship with education ^33^, while the relationship is positive and linear (prevalence increases with increase in educational level) in studies from LMICs ^31^. Further, some studies from India and Kerala failed to establish any relationship between educational status and multi-morbidity ^34, 35^. Our study indicates a higher prevalence in individuals with relatively low education levels. However, on adjustment for other socio-demographic characteristics, individuals with less than primary school education had lower propensity for multi-morbidity as compared to individuals with more than primary school level education. Under-reporting of self-reported chronic conditions, especially in the low education group ^36^, often results in positive associations of education with multi-morbidity. We recommend further investigation to profile the pattern of multi-morbidity according to attained educational status and related inequality.

### Multi-morbidity pattern and their implications

The most common pair of coexisting chronic conditions in our study was diabetes and hypertension in males and females. This finding is consistent with the results of the study conducted by Singh and colleagues ^27^. The second most common pair was hypertension and depression for females, while it was hypertension and ischemic heart disease for males. Similarly, the most common triad in females was diabetes, hypertension and depression, while in males it was diabetes, hypertension and ischemic heart disease. Higher health care utilization and poor quality of life ^20^ in individuals with multiple chronic conditions calls for integrated prevention and control strategies to address the burden of multi-morbidity.

### Strengths and limitations

In our survey, we achieved a high response rate (95%) and incorporated measures for active screening of under-diagnosed chronic conditions such as hypertension, diabetes and depression. Further, a medically qualified primary care physician reviewed the medical records of all participants to confirm the self-reported status of other chronic conditions. However multi-morbidity assessment was done by simple counting and the severity of the conditions were not taken in to consideration. Random blood sugar for identification of individuals with diabetes may have under-estimated the true burden of diabetes. Association observed in our study does not imply causality due to the cross-sectional study design.

## Conclusion

Multi-morbidity is a major public health problem in Kerala that affects almost half of adult individuals in the productive age group and seven out of ten households. Diabetes, hypertension, depression and ischemic heart disease are the most common co-existing conditions. However, the pattern of multi-morbidity is different across gender and other socio-economic groups. Future research is recommended to identify the progression of single chronic conditions to multi-morbidity over the life-course using prospective study designs.

## Data availability

### Underlying data

https://doi.org/10.6084/m9.figshare.12494681.v4

Figshare: Prevalence and patterns of multi-morbidity among 30–69 years old population of rural Pathanamthitta, a district of Kerala, India: A cross-sectional study. https://doi.org/10.6084/m9.figshare.12494681.v4 ^16^.

This project contains the following underlying data:

- Multilatest v2 - Copy.xls (demographic and clinical data for all participants)
- Codes used in multilatest v2-converted.pdf (data dictionary)

### Extended data

Figshare: Prevalence and patterns of multi-morbidity among 30–69 years old population of rural Pathanamthitta, a district of Kerala, India: A cross-sectional study. https://doi.org/10.6084/m9.figshare.12494681.v4 ^16^.

This project contains the following extended data:

- Interview schedule english version 3-converted.pdf (interview schedule in English)
- Table for interview english version 1-converted.pdf (interview schedule table in English)
- Interview schedule malayalam version2.pdf (interview schedule in Malayalam)
- Table for interview malayalam version1-converted.pdf (interview schedule in Malayalam)

Data are available under the terms of the Creative Commons Attribution 4.0 International license (CC-BY 4.0).
