# Supplementary material for: Prevalence and patterns of multi-morbidity in the productive age group of 30-69 years: A cross-sectional study in Pathanamthitta District, Kerala
Source: Wellcome Open Res. 2020 Dec 15;5:233. Originally published 2020 Oct 8. [Version 2] doi: 10.12688/wellcomeopenres.16326.2 (PMC7658726; doi:10.12688/wellcomeopenres.16326.2)
Supplement: Peer review history

# Peer review: Reviewer response for version 2

**DOI:** 10.21956/wellcomeopenres.18178.r41845

**Published:** 2020-12-21

**Authors:** Pallab K Maulik

**Affiliations:** 1 George Institute for Global Health, New Delhi, Delhi, India

Is the work clearly and accurately presented and does it cite the current literature?

Yes

If applicable, is the statistical analysis and its interpretation appropriate?

Yes

Are all the source data underlying the results available to ensure full reproducibility?

Yes

Is the study design appropriate and is the work technically sound?

Partly

Are the conclusions drawn adequately supported by the results?

Yes

Are sufficient details of methods and analysis provided to allow replication by others?

Yes

Reviewer Expertise:

Mental health

I confirm that I have read this submission and believe that I have an appropriate level of expertise to confirm that it is of an acceptable scientific standard.

# Peer review: Reviewer response for version 1

**DOI:** 10.21956/wellcomeopenres.17948.r41074

**Published:** 2020-11-11

**Authors:** GK Mini

**Affiliations:** 1 Global Institute of Public Health, Ananthapuri Hospitals and Research Institute, Trivandrum, Kerala, India

**Comments and suggestions:**

 The study is well conducted and should be of interest to the readership, my congratulations to the authors for picking up the topic, multi-morbidity. However, there are some minor problems that need to be addressed by the authors.

**Abstract:**

 The abstract is concise, specific, representative of the article, and in the correct form, which accurately summarizes the essential information.

- The first sentence in the abstract, “Multi-morbidity is the coexistence of multiple chronic conditions in individuals and families,” is slightly different from the definition of multi-morbidity given in the methodology. Better to avoid “families” from abstract.
- The objective is not given in the abstract.

**Methods:**

 The methodology of the study is thorough and presented clearly. The researchers have done a good job describing the study population, data collection, definitions of terms used, and sample size.

- Since all the eligible participants in the selected households were interviewed, there is a possibility for the clustering of multiple respondents within the houses, which is not addressed in the analysis.
- The details of the number of households surveyed were not given in the abstract. This is important in the context of the discussion on multi-morbidity assessment at the household level.
- Only elderly participants were asked about their living arrangements. This needs further explanation.
- Under the sub-title ‘Data Collection,’ “Malayalam” can be put in italics and bracket (local language), helping international readers.
- The justification for selecting the 11 listed chronic conditions in the study were not given.
- There is no mention of the type of treatment for the selected diseases.
- Patients with multi-morbidity are at increased risk for polypharmacy. Since the authors verified the medical records, it would have been easy for them to get details of medication and the treatment from different systems.

**Results:**

 The results are clearly described. Some points for consideration are as follows:

- The study used multi-stage cluster sampling. Correction for clustering was not performed in data analysis.
- Depression was measured using PHQ-9. However, no detail on the prevalence of depression is presented in the manuscript.
- The results derived from the logistic regression analysis presented in Table 3, age standardization would have helped estimate the true prevalence of multi-morbidity in this population, which would also help the readers to compare results from other studies.
- The classification of employment status presented in Table 1 & Table 3 is different. I’d suggest making these consistent.
- In Table 2, the authors gave details of common pairs of chronic conditions. However, I couldn’t find the prevalence of individual diseases anywhere in the manuscript.
- In Table 3, in employment classification, better to club the ‘salaried’ with the first category is ‘daily wage/self-employed’ so that it becomes two categories as employed and not employed.
- In Table 3, marital status classification needs to be verified, specifically the term ‘currently not married.’
- Prevalence of the recommended level of physical activity was given in the manuscript. It would be better to analyze the recommended level of physical activity with multi-morbidity considering the association of physical activity with decreased risk of chronic conditions and mortality.     

**Discussion:**

 The discussion section is very well presented as it covers all of the components associated with the study. The conclusion is based on the study's findings, and the recommendations made would help future related studies.

- In the results section, the association of menopause and multi-morbidity was given, and there is a significantly increased prevalence of multi-morbidity among those who reached menopause. However, no further discussion is seen in this association. Higher age of menopause women may be considered in this case. 

Is the work clearly and accurately presented and does it cite the current literature?

Yes

If applicable, is the statistical analysis and its interpretation appropriate?

Yes

Are all the source data underlying the results available to ensure full reproducibility?

Yes

Is the study design appropriate and is the work technically sound?

Yes

Are the conclusions drawn adequately supported by the results?

Yes

Are sufficient details of methods and analysis provided to allow replication by others?

Yes

Reviewer Expertise:

Chronic Disease Epidemiology, Quantitative Research Methods

I confirm that I have read this submission and believe that I have an appropriate level of expertise to confirm that it is of an acceptable scientific standard.

# Response

**Published:** 2020-12-09

**Authors:** Panniyammakal Jeemon

**Affiliations:** Sree Chitra Tirunal Institute for Medical Sciences and Technology, Trivandrum, India

Thank you very much for the positive comments. We have included our detailed response to the comments in the section below: 

***Comment 1*: *The first sentence in the abstract, “Multi-morbidity is the coexistence of multiple chronic conditions in individuals and families,” is slightly different from the definition of multimorbidity given in the methodology. Better to avoid “families” from abstract. ○ ○ The objective is not given in the abstract.***

 As per the suggestion we have remove the word "families" from the abstract. We have also added the main objective of the study in the abstract. 

***Comment 2*a: *Since all the eligible participants in the selected households were interviewed, there is a possibility for the clustering of multiple respondents within the houses, which is not addressed in the analysis*.**

 We had included all eligible participants to assess the multi-morbidity burden at the household level. The household level multi-morbidity is a new concept and has important implications in prevention and control of chronic conditions. We had included 193 households (clusters) in the study and used a sandwich variance estimator in the regression model. 

***Comment 2c: Only elderly participants were asked about their living arrangements. This needs further explanation*.**

 We were interested in generating some pilot data on the relationship between living arrangements and multi-morbidity burden in the elderly population. It was not part of the main objective of the study. 

***Comment 2d:Under the sub-title ‘Data Collection,’ “Malayalam” can be put in italics and bracket (local language), helping international readers*.**

*We have inserted a bracket and mentioned that it is the local language in Kerala.*

***Comment 2e: The justification for selecting the 11 listed chronic conditions in the study were not given.*** ****

 This is purely because of pragmatic reasons and based on consensus opinion from experts in the field. 

***Comment 2f: There is no mention of the type of treatment for the selected diseases. Patients with multi-morbidity are at increased risk for polypharmacy. Since the authors verified the medical records, it would have been easy for them to get details of medication and the treatment from different systems.***

 Yes. As the reviewer correctly pointed out the patients with multi-morbidity are at increased risk of poly-pharmacy. Although we collected details of medications, we could not analyze the data as there was no uniformity regarding the type of treatment especially between various systems of medicine. We will need to discuss this with experts in alternative systems of medicine to complete the data analysis. 

***Comment 3a:The study used multi-stage cluster sampling. Correction for clustering was not performed in data analysis*.**

 We had included 193 households (clusters) in the study and used a sandwich variance estimator in the regression model. 

***Comment 3b:Depression was measured using PHQ-9. However, no detail on the prevalence of depression is presented in the manuscript***

 Prevalence of individual conditions are also presented in Figure 2. 

*Comment 3c:* *The results derived from the logistic regression analysis presented in Table 3, age standardization would have helped estimate the true prevalence of multi-morbidity in this population, which would also help the readers to compare results from other studies* **.**

 We agree that age-standardization would have helped for comparison of data with other studies. However, since it was a study conducted in one of the districts in Kerala, it may not be useful even if we age standardize to the world standard or Indian population. 

***Comment 3e:In Table 2, the authors gave details of common pairs of chronic conditions. However, I couldn’t find the prevalence of individual diseases anywhere in the manuscript.***

 Prevalence of individual conditions are also presented in Figure 2. 

***Comment 3f:In Table 3, in employment classification, better to club the ‘salaried’ with the first category is ‘daily wage/self-employed’ so that it becomes two categories as employed and not employed.***

 Thank you for this suggestion. We have made the changes as per the suggestion. 

***Comment 3g:In Table 3, marital status classification needs to be verified, specifically the term ‘currently not married*.’**

 We have added a footnote to explain the definition of this variable. 

***Comment 3h:Prevalence of the recommended level of physical activity was given in the manuscript. It would be better to analyze the recommended level of physical activity with multi-morbidity considering the association of physical activity with decreased risk of chronic conditions and mortality.***

 The limitation of the study design made the assessment difficult as many of the participants started doing the recommended level of physical activity only after they have been diagnosed with one or more of the chronic conditions.

***Comment 4*: *In the results section, the association of menopause and multi-morbidity was given, and there is a significantly increased prevalence of multi-morbidity among those who reached menopause. However, no further discussion is seen in this association. Higher age of menopause women may be considered in this case.***

 As the reviewer pointed out, there was a higher prevalence of multi-morbidity among females who attained menopause. However, after adjusting for age this difference was marginal.

# Peer review: Reviewer response for version 1

**DOI:** 10.21956/wellcomeopenres.17948.r40796

**Published:** 2020-10-26

**Authors:** Pallab K Maulik

**Affiliations:** 1 George Institute for Global Health, New Delhi, Delhi, India

It is good paper ascertaining multimorbidities in the south Indian state of Kerala (one district). Overall, I have a few observations which are now not modifiable, but something that needs to be addressed in the discussion:

1. The district chosen seems one of the more extreme districts of Kerala in many ways as already indicated by the author and Kerala itself is one of the odd states in India - health indicator wise. This does bring into question issues about generatlizability of the data and more discussion needs to be given to that.
2. The study excluded pregnant and lactating women. Not sure about the rationality as it definitely takes out the possibility of perinatal depression and some other pregnancy related health issues.
3. There is no information about the temporality of the occurrence of the diseases. If that data was not collected, one needs to include that in the limitations and comment on it.  
4. While the study may not be powered to provide gender-based analysis, some preliminary indications of trends could be useful, especially given that the results were affected by gender.
5. The author may want to look into the Academy of Medical Science, UK report on multimorbidity.  

Is the work clearly and accurately presented and does it cite the current literature?

Yes

If applicable, is the statistical analysis and its interpretation appropriate?

Yes

Are all the source data underlying the results available to ensure full reproducibility?

Yes

Is the study design appropriate and is the work technically sound?

Partly

Are the conclusions drawn adequately supported by the results?

Yes

Are sufficient details of methods and analysis provided to allow replication by others?

Yes

Reviewer Expertise:

Mental health

I confirm that I have read this submission and believe that I have an appropriate level of expertise to confirm that it is of an acceptable scientific standard.

# Response

**Published:** 2020-12-09

**Authors:** Panniyammakal Jeemon

**Affiliations:** Sree Chitra Tirunal Institute for Medical Sciences and Technology, Trivandrum, India

We thank the reviewer for their positive comments. Our detailed response to the comments are given below; 

***Comment 1*: *The district chosen seems one of the more extreme districts of Kerala in many ways as already indicated by the author and Kerala itself is one of the odd states in India – health indicator wise. This does bring into question issues about generalizability of the data and more discussion needs to be given to that*.**

*We agree that the results may not be generalisable to other population with different population structure. We have therefore given the name of the district and the state in the title itself. Further, we have described the socio-demographic pattern of the selected district in the study settings.*

***Comment 2:*** ***The study excluded pregnant and lactating women. Not sure about the rationality as it definitely takes out the possibility of perinatal depression and some other pregnancy related health issues.***

*We agree that exclusion of pregnant and lactating women would have underestimated the  prevalence of multi-morbidity. However, some of the chronic conditions like depression may be relatively transient (not very chronic) in this group of participants. Further, it is a standard methodology to exclude pregnant women from similar surveys.*

***Comment 3: There is no information about the temporality of the occurrence of the diseases. If that data was not collected, one needs to include that in the limitations and comment on it.***

 Yes. As the design of the study is cross-sectional, temporality cannot be ensured. We accept it as a limitation of this study design.

***Comment 4*: *While the study may not be powered to provide gender-based analysis, some preliminary indications of trends could be useful, especially given that the results were affected by gender.***

 We have presented the common pairs of chronic conditions stratified by gender in Table 2. 

***Comment 5:The author may want to look into the Academy of Medical Science, UK report on multi-morbidity.***

*Thank you for this suggestion. We were motivated to conduct this assessment after reading the Academy of Medical Science, UK report on multi-morbidity.*

**

*Once again we thank the reviewer for his positive comments.*

**
